# Opportunity Finding by Nascent Entrepreneurs: Accidental or Purposeful?

**DOI:** 10.3389/fpsyg.2020.592994

**Published:** 2021-01-28

**Authors:** Rongji Zhou, Sibin Wu

**Affiliations:** ^1^Hengyang Normal University, Hengyang, China; ^2^The University of Texas Rio Grande Valley, Edinburg, TX, United States

**Keywords:** nascent entrepreneurs, opportunity finding, active search, satisficing search, wealth aspiration, need for achievement, motivation

## Abstract

**Purpose:**

This research studies an important, but relatively unexplored entrepreneurial aspect: motivation and aspiration on opportunity finding/discovery.

**Design/Methodology/Approach:**

This study surveyed 230 nascent entrepreneurs on their opportunity finding behavior. A poisson regression and a logistic analysis were conducted to discover the relationship between motivation/aspiration and opportunity search behavior.

**Findings:**

Motivation and aspiration interact to influence active search in a positive way. However, only willingness to become an entrepreneur is found to search for opportunities purposefully.

**Research Limitations/Implications:**

Participants of the research are from a Midwest state in United States. Future research may collect sample from more and larger areas.

**Practical Implications:**

Bankers may use entrepreneurial opportunity search behavior as one criterion determining if to fund a person or not.

**Originality/Value:**

This article answers the call to study motivation/aspiration on opportunity finding (Yitshaki and Kropp, 2018; Murnieks et al., 2020). It is one of the first studies to explore the above relationship.

## Introduction

Stevenson argues that the main reason a potential entrepreneur doesn’t pursue an opportunity is not, as is often claimed, lack of money. Instead, “what is stopping them is that what they’ve got is not truly an opportunity – it is a bad idea” (2003: 5). Indeed, opportunity finding stands at the center of entrepreneurship (e.g., Shane and Venkataraman, 2000), representing the most distinctive and fundamental entrepreneurial behavior (Yitshaki and Kropp, 2016). Once individuals possess worthwhile opportunities, they can begin the entrepreneurial process. And often, if individuals can identify true opportunities, others will provide resources so those opportunities can be exploited (Shane and Cable, 2002; Joardar et al., 2014).

Yet, given the importance of opportunity finding for entrepreneurs, studies focusing on the opportunity finding process are rare, and have produced equivocal results (Yitshaki and Kropp, 2018). This has led to two quite different perspectives on opportunity finding. Shane (2000), on the one hand, argues that people can and will discover entrepreneurial opportunities without actively searching for them. He found that entrepreneurs were surprised by the opportunities that they discovered, and that their opportunity discovery process was often attributed to serendipity. This situation is well represented by the case of Yolanda Zambrano. She came from Colombia to the United States to marry her husband in Worcester, MA in 1990. At the beginning, she spent most of her time learning English, in the hope of continuing her college studies. But, 2 years later, she seized the chance to help out an acquaintance in a local travel reservations office. Just 3 weeks later the owner, who was a fellow Colombian, offered to sell her the business. Surprising even herself, Ms. Zambrano took the bait. “I didn’t have the money or the experience. I had never even thought about this,” she says. “But then I thought, well, why not?” (Mangi, 2001). Within 5 years, Ms. Zambrano built the Alpha Travel Agency into a thriving $5.5 million business.

Not everyone agrees that opportunity often can be found by surprise, however. Drucker (1998) argues that most innovations – specific functions of entrepreneurship – result from the purposeful search for opportunities. According to Drucker, successful innovation results from careful analysis of the sources of new opportunities. Thus, as found to be the case in several studies (e.g., Kraus et al., 2017; Yitshaki and Kropp, 2018; Murnieks et al., 2020), entrepreneurs should employ proactive attentiveness (alertness) to the market environment in order to find desirable and feasible ideas (Neneh, 2019). This view is well represented by the case of Masayoshi Son. Son started searching for ideas when he was attending graduate school at UC-Berkeley (Webber, 1992). When he got back to Japan, he spent 2 years in libraries and bookstores looking for business ideas. He then made a list of the 40 new ideas that he had developed, and he evaluated each one based on 25 success factors. Finally, he chose the idea he would pursue. He did not want to start just any business, but instead wanted to start a business that would succeed for the next 30–50 years. He launched SOFTBANK, and by the early 1990s became one of the richest people in Japan. Nowadays, he is considered as the most powerful person in Silicon Valley (Brooker, 2019).

Ms. Zambrano and Mr. Son are both successful entrepreneurs. However, they differ greatly in their opportunity searching behavior. First, Mr. Son actively searched for opportunities and he examined many ideas, while Ms. Zambrano did not look for ideas at all. Second, Mr. Son selected the best idea out of many ideas that he considered, while Ms. Zambrano took the only idea available and put it into action. Third, Mr. Son systematically and carefully assessed all his ideas before exploiting the best one, while Ms. Zambrano used her intuition. We label entrepreneurs like Mr. Son purposeful entrepreneurs, and those like Ms. Zambrano accidental entrepreneurs; they differ in whether they actively search for opportunities and in whether they satisfice when selecting an opportunity.

In this study, we take a step toward better understanding questions such as: Do potential entrepreneurs “find” a terrific opportunity that compels them to start a venture, without extensive search? Or do most nascent entrepreneurs strive to optimize during opportunity search? Or is it often some combination of satisficing and optimizing behaviors? We also seek to understand better how characteristics of prospective entrepreneurs – specifically, wealth aspirations and motivation to become an entrepreneur – might influence their opportunity finding processes (Turner and Pennington, 2015).

By answering the above questions, we intend to make the following contributions. First, motivation plays a very important role in entrepreneurial process and success (Yitshaki and Kropp, 2016; Murnieks et al., 2020). Nonetheless, research on the impact of motivation on entrepreneurship has been fragmented (Shane et al., 2003; Murnieks et al., 2020). Such fragmentation can be attributed to four possibilities that (1) motivations do not matter, (2) they work with other variables together, (3) wrong motivations have been used, and (4) motivations may be more crucial to the early stages of venture creation (Baum et al., 2001; George et al., 2016). The current study bases on the assumption that motivations matter to entrepreneurship (Frese and Gielnik, 2014; Murnieks et al., 2020). In addition, the research examines the right motivations (e.g., need for achievement) that have been consistently proven to be significant factors to the entrepreneurial process (e.g., Robichaud et al., 2001; Yitshaki and Kropp, 2018). Further, our research studies the interaction effect of motivation and wealth aspiration in the initiation stage of the entrepreneurial process when motivations as a desire for self-starting are especially salient (Frese and Gielnik, 2014). We conduct the research to answer recent calls to address the issues on motivations and opportunity finding (Yitshaki and Kropp, 2018; Murnieks et al., 2020).

Second, previous scholars have diverged on how nascent entrepreneurs search for opportunities (Korsgaard, 2013). On one hand, entrepreneurs may find their opportunities by accident without active search (Shane, 2000). On the other hand, entrepreneurs’ opportunities can also be found through systematic search (Patel and Fiet, 2011). It is even more puzzling that both streams of research find that prior knowledge influences how entrepreneurs search for opportunities (George et al., 2016). The two seemingly contradicting views hold two common assumptions that opportunity has been evaluated and selected, and that opportunity discovery is a one-dimension phenomenon. Such assumptions can be challenged. For example, Tang et al. (2012) depict opportunity discovery as containing three dimensions: search, association, and evaluation/judgment. While entrepreneurs may need prior knowledge to make the right connections and best judgment to evaluate and choose the best opportunity to pursue, they need to be motivated to find good opportunities (George et al., 2016). Therefore, our study contributes by reconciling the two diverging views by focusing on the relationship between motivation/aspiration and opportunity finding (Murnieks et al., 2020). Our research shows that aspiration can lead to active search while need for independence has a negative effect on search intensity. Third, our study makes empirical contribution to entrepreneurship opportunity research. Specifically, we view opportunity search as two dimensions: active vs. passive (Tang et al., 2012), and satisficing vs. maximizing (March and Simon, 1958). Our approach answers the call for further refined measurement on opportunity recognition (George et al., 2016).

In the sections that follow, we first argue that entrepreneurs may undertake differing degrees of active search for opportunities, and that during the search process they may tend to satisfice or try to optimize with their opportunity choice. Thus, both views about entrepreneurs’ opportunity search are valid, and both are components of the whole picture. We then describe our field study of the opportunity search behaviors of nascent entrepreneurs, and present its results. Finally, we outline the implications of our work, for entrepreneurs and for future research in opportunity finding.

## Literature Review and Hypothesis Development

### Entrepreneurial Opportunity

Entrepreneurship is at the nexus of individuals and opportunities (Shane and Venkataraman, 2000). Given that opportunity has been viewed as the distinctive domain of entrepreneurship research (Shane, 2012), it is not surprising that opportunity research has received much attention (Tang et al., 2012; Yitshaki and Kropp, 2018).

Entrepreneurship can be viewed as a process where individuals may engage in opportunity search/finding (Shook et al., 2003), opportunity discovery (George et al., 2016), opportunity evaluation (Haynie et al., 2009), and opportunity exploitation (Choi and Shepherd, 2004). Even though entrepreneurial process may not be linear, there are distinctions across those activities. For example, opportunity exploitation decision may be made based on the availability of potential resources (Choi and Shepherd, 2004) while evaluation activity may be initiated by social capital (Brännback and Carsrud, 2016). As stated above, opportunity results from ideas and opportunity finding is the first necessary step of the entrepreneurial process (Stevenson, 2003). Only with a set of ideas to consider, will entrepreneurs be able to find new ways to make profit through making new connections between means and ends to create new products or services (Shane, 2012; Chetty et al., 2018). However, opportunity finding research has bee lacking. Our research fills such a research void.

### Theoretical Framework

Need-motive-value theory can be used to explain the tendency in an entrepreneurial context. According to this theory, individuals differ in their needs and values. Basic and innate human needs drive people to engage in activities which satisfy their needs. According to Alderfer (1969), people’s tendency to meet unsatisfied needs mobilize people’s behavior to the direction that those needs can be finally satisfied.

We can also use the achievement theory to explain entrepreneurial persistence. McClelland (1961) believed that human beings’ behavior is guided by need for achievement. Such a motive regulates human action over the long term, hence likely to lead to search behavior (George et al., 2016). People high in need for achievement tend to believe that they have the control over the outcome of their behavior and that they also can have the right feedback about their progress toward their goal. More important, such motives play a key role in entrepreneurial occupation. Both need-motive-value theory and need for achievement theory indicate that entrepreneurial needs are important prior factors leading to opportunity search. According to motivation theory, satisfied needs resolve intensity issues and hence should motivate entrepreneurs (Robbins, 2001).

### Motivation, Aspiration and Opportunity Search

Nascent entrepreneurs search for opportunities differently, depending on their perceptions about the ideas available to them and their goals, ability and knowledge. When entrepreneurs believe that many desirable opportunities are available, and when they are confident that they have the knowledge and skills to make the opportunities realized, they tend to take their time and do formal, detailed and systematic analysis – i.e., they try to optimize (Daft and Weick, 1984). When entrepreneurs believe an opportunity will be short-lived, or if they do not have an ambitious goal, they tend to make quick decisions using heuristic rules, intuition, and subjective judgment – i.e., they satisfice (Gilbert-Saad et al., 2018). Thus, we must examine both motivation and aspiration in order to understand opportunity search behaviors (Sirec and Mocnik, 2013; Yitshaki and Kropp, 2018).

Motivation to be an entrepreneur matters (Stevenson and Jarillo, 1990), and it matters especially in the opportunity searching behavior (Baum et al., 2001; Shane et al., 2003; Yitshaki and Kropp, 2018). We also argue, however, that motivation may not work alone in entrepreneurial opportunity discovery process. High aspiration levels for a future venture, for example, demand that entrepreneurs consider more opportunities and act on the best one, while low aspiration levels (for example, to maintain certain life style) lead toward discovery of one satisfactory opportunity and acting upon it. How entrepreneurs search for their opportunities is then related to both motivation and aspiration level (March and Simon, 1958; Goel and Karri, 2020). We address each variable next in turn.

#### Motivation

Research on the importance of motivation in new venture studies has been inconsistent and fragmented (e.g., Baum et al., 2001; Shane et al., 2003). Individuals are driven to be entrepreneurs by needs. Need theories indicate that needs reflect a state of dissatisfaction (March and Simon, 1958; Alderfer, 1969), and that dissatisfied individuals are motivated to search for alternatives to satisfy their needs (Daft, 1997). Prospective entrepreneurs are often unsatisfied with their current work situations, due to lack of freedom or lack of challenge (Morrison, 2001). Their dissatisfaction increases their motivation to become their own bosses, which spurs opportunity search. Research has found three important entrepreneurial motivation, reflecting the needs for: independence/autonomy, achievement, and willingness to become an entrepreneur (Robichaud et al., 2001; Stewart and Roth, 2007; Wiklund et al., 2017). Each is addressed next.

First, the need for autonomy has been cited as one of the most important motivations for entrepreneurial orientation (e.g., Osborne, 1995; Wales, 2016). Autonomy is a necessity for new-entry activity and hence serves a key dimension for nascent entrepreneurs (Wales, 2016). Autonomy refers to the independent action of an individual in bringing forth an idea or vision and carrying it through a completion. According to need theories, the need for autonomy or independence is the result of dissatisfaction from lack of freedom (Lawler, 2010). To satisfy the need, then, is to reduce the dissatisfaction and satisfying the need for autonomy. Therefore, when an individual feels the need to be independent, the first thing for him/her to do is to look for alternative employment or becoming an entrepreneur. Being an entrepreneur grants a person this freedom and satisfies the need. If a nascent entrepreneur’s need for independence is strong, he/she will likely intensify his/her search for entrepreneurial opportunities, and will likely find more ideas. Thus:

H1: The number of ideas that an entrepreneur finds in a period of time is positively related to the need for independence.

Second, the need for achievement is defined as behavior toward competition with a standard of excellence (Chen et al., 2012). This need motivates individuals to face challenges (Lee, 1996). The entrepreneurial process is full of uncertainty and risks and, correspondingly, challenges (e.g., Koudstaal et al., 2016). One has to come out with an attractive idea, and then obtain the capital needed to carry out the idea to become a successful entrepreneur. Nascent entrepreneurs with strong need for achievement likely will look more vigorously for entrepreneurial opportunities, so as to become entrepreneurs, and will likely consider more ideas. Thus:

H2: The number of ideas that an entrepreneur finds in a period of time is positively related to the need for achievement.

These needs may lead to the drive to become entrepreneurs (Robbins, 2001), but they may not be sufficient to drive an individual to take on an entrepreneurial career. This requires a more specific entrepreneurial motivation, i.e., a person has to be willing to take on the risks associated with entrepreneurial activities, often at the sacrifice of some family security. This willingness is said to be the essence of entrepreneurship (Stevenson and Jarillo, 1990; Yurtkoru et al., 2014), meaning that individuals are willing to test something new and risky. Therefore, willingness is an important variable in the opportunity search process; those who are willing to devote more time to the entrepreneurial process likely will actively search for opportunities, and will likely find more ideas to consider. Thus:

H3: The number of ideas that an entrepreneur finds in a period of time is positively related to willingness to become an entrepreneur.

#### Wealth Aspiration Level

Personal wealth goals determine not only the target size of the businesses that entrepreneurs intend to launch, but also their opportunity searching process (Westhead and Wright, 1998). Gimeno et al. (1997) view entrepreneurs as choosing between establishing a venture and obtaining alternative employment. They reason that entrepreneurs would terminate their businesses if the expected utility of alternative employment minus the cost inherent in switching exceeds the expected utility of remaining in the venture. They argue that entrepreneurs’ objectives play a dominant role in dictating the direction of entrepreneurial behavior (Delmar and Wiklund, 2008; Mocinik and Sirec, 2016).

High wealth aspiration levels for the future venture demand that entrepreneurs consider more alternatives, developing more ideas through active search and then carefully acting on the most desirable one in order to meet their aspirations. Individuals with low aspiration levels, on the other hand, need only to find a few alternatives to meet their goals (Bhide, 1994). Thus:

H4: The number of ideas that the entrepreneurs find in a period of time is positively associated with wealth aspiration level.

As entrepreneurs build a stock of ideas, they must decide when to stop, evaluate and choose the best from among their alternatives (McGrath and MacMillan, 2000). Some entrepreneurs may arrive at one acceptable idea and act upon it (Stevenson and Jarillo, 1990), while others may pursue multiple ideas and use optimizing criteria to select the best one (Gaglio and Katz, 2001). Such “optimize or satisfice” decisions are affected by aspiration level (Herron and Sapienza, 1992). Thus, the entrepreneur’s wealth aspiration level must be included in a model of the opportunity finding process. Entrepreneurs compare possible outcomes relative to some reference or aspiration level to determine their desirability (Uy et al., 2013). If the aspiration level is low, it becomes easy to find a satisfactory and desirable alternative (Simon, 1979), rendering systematic analysis unnecessary. Hence, low aspiration levels likely contribute to satisficing in the opportunity finding process. Conversely, high aspiration levels call for active search, and for consideration of more ideas, because ambitious endeavors require significant capital and hence need to be better researched and planned (Bhide, 1994). Thus:

H5: Satisficing during opportunity finding is negatively associated with wealth aspiration level.

#### Motivation, Wealth Aspiration and Opportunity Search

We now argue that motivation also works jointly with wealth aspiration levels in affecting opportunity search (Murnieks et al., 2020). As argued earlier, aspiration levels influence active search (Mocinik and Sirec, 2016); specifically, aspiration level is positively related to the number of ideas considered and that aspiration level is negatively related to satisficing. In sum, low aspiration entrepreneurs tend to search passively for opportunities, consider fewer alternatives, and make quick idea selection decisions.

These hypothesized relationships, however, are moderated by entrepreneurial motivation. Overall, a high aspiration level calls for active search and the consideration of many alternatives. Thus, the relationship between aspiration level and search is positive. This relationship will be strongest for highly motivated individuals (George et al., 2016), such as entrepreneurs who want to be independent very badly. They want to be entrepreneurs more eagerly, so as to satisfy their need, and they therefore are very aggressive in considering ideas, and particularly so when they have high aspirations (Hills et al., 1999). However, for less motivated entrepreneurs, even when aspiration becomes greater, their searching behavior tends to be less active. They may be vacant entrepreneurs and they tend to seek fewer ideas (Chatman and Cha, 2003). We can also argue from the goal setting perspective. For example, Gartner et al. (1992) linked high aspirations with future survival of entrepreneurial firms. Baum et al. (2001) found that high growth aspiration CEO founders set for their businesses are positively related to the actual venture growth. Hence, the hypothesized relationship between the number of ideas considered and wealth aspiration levels is moderated by entrepreneurial motivation, as follows.

H6: A change in wealth aspiration will have a greater effect on the number of ideas considered when the individual has a high motivation to become an entrepreneur.

As in hypothesis 5, the relationship between satisficing and wealth aspiration level is negative. However, highly motivated entrepreneurs who are searching for challenges and want to grow and learn will do their very best to fulfill their expectation (Matthews and Human, 2000). Less motivated entrepreneurs, no matter what their wealth aspiration level, may simply take any ideas that are presented to them (Shane, 2000). Their satisficing tends to be high in any scenario because they may be vacuous entrepreneurs (Chatman and Cha, 2003). For highly motivated entrepreneurs when aspiration is low, they can rather easily find ideas that satisfy their needs, and that makes their selection of ideas immediate so they can take quick action. For highly motivated entrepreneurs when their aspiration is high, they must search more actively for ideas to meet the high aspiration, and that tends to delay their actions of exploitation. Research has shown that bigger and hairy goals may help stimulate themselves to persist even in face of obstacles (Collins and Porras, 1996). Entrepreneurs with strong needs accompanied by high goals may strive to achieve the goals for success. Hence, the hypothesized relationship between satisficing and wealth aspiration is moderated by entrepreneurial motivation, as follows.

H7: A change in wealth aspiration will have a greater effect on satisficing when the individual has a higher motivation to become an entrepreneur.

## Materials and Methods

### Sample

We collected data from prospective entrepreneurs attending seminars on basic venturing offered through four local agencies in a Midwestern United States city: a Service Corps of Retired Executives (SCORE) chapter, a university Small Business Development Center, a Woman’s Business Initiatives Corporation, and a Hmong American Friendship Association. A survey questionnaire about motivation, aspiration and opportunity search was designed, pilot tested and revised, and then was administered at the beginning of seminar sessions. We visited 16 seminars and workshops. Our study’s high participation rate of 72% yielded 230 completed surveys.

### Dependent Variables

Supplementary Appendix A provides a brief description of all variables and their coding.

#### Number of Ideas Considered

This item asked participants how many different business ideas they had considered in the past 3 months. Hills et al. (1999) used a similar item, but with a 5-year span. Since we focus on how nascent entrepreneurs proceed toward and pursue opportunities, and given that the average time for an entrepreneur to start from an idea to a registered sale is about 1 year, we think using a 3-month period captures the information sought (Reynolds and Miller, 1992). The assumption of normality necessary when using linear regression was violated for this variable. Several attempts at transformation failed to satisfy tests for normality, so we ultimately used the non-linear analysis of Poisson regression.

#### Satisficing Search

Satisficing differs from optimizing in that satisficers consider fewer options. Satisficers tend to spend less time on formal analysis; when they find an opportunity they judge good enough, they begin the exploitation process (Burke and Miller, 1999). We categorized some nascent entrepreneurs as being satisficers based on three criteria. First, they considered relatively fewer ideas (less than or equal to the median of 2); second, they had selected an idea to act on; and third, they reportedly had committed at least one of the three discriminating activities identified by Carter et al. (1996) research: they had purchased equipment, developed a model or prototype, or asked for external funds. Non-satisficers are those who either considered many ideas, or had not chosen an idea to pursue yet, or had selected an idea to pursue, but had not taken any serious effort to realize the idea. We coded satisfiers as “1” and non-satisficers as “0.”

### Independent Variables

#### Needs for Independence and Achievement

We adapted the motivation instruments developed by Robichaud et al. (2001), who had based their work partially on a study by Kuratko et al. (1997). Based on previous theories that entrepreneurs tend to be independent (Osborne, 1995) and long for achievement (Gartner, 1988), we selected the three most reliable items for each of two dimensions: autonomy/independence and intrinsic motivation. Kolvereid (1992) used a different instrument with more dimensions, but the two motives identified in his study are similar to that used by Robichaud and associates. We added one item from Kolvereid’s work for each of the two dimensions that were common in both studies, and merged these items with the selected items from Robichaud et al. (2001). This yielded four items on independence and four items on achievement. All items are measured on a 1–5 scale, with 1 as strongly disagree and 5 as strongly agree. A principal component factor method and varimax rotation produced a two-factor structure based on an eigenvalue test and a Scree plot of eigenvalues. The eight items are: I need to make my own decisions, to have greater flexibility for life, to maintain my personal freedom, to be my own boss (independence), to meet the challenge, to continue learning, personal growth, and to prove that I can succeed (achievement). Cronbach alphas greater than 0.80 for both factors corroborate the reliability.

#### Willingness to Become an Entrepreneur

We measured entrepreneurial motivation, the desire to become an entrepreneur, using the willingness script scale by Mitchell et al. (2000). It contains four items measuring how likely an entrepreneur is to explore new and challenging opportunities. Each item contains two choices, with (a) indicating the desire to try new things and (b) indicating the propensity to maintain the *status quo*. For example, respondents are asked if they are more comfortable with (a) new situations, or (b) familiar territory. A choice of (a) is coded as one and (b) as zero, and the scores sum (Mitchell et al., 2000).

#### Wealth Aspiration

We used the nascent entrepreneurs’ expectations about the future sales for the fifth year in business to assess their levels of wealth aspiration, following the practice of Matthews and Human (2000) and Delmar and Davidsson (1999). Aspiration level refers to the borderline between perceived success and failure (Greve, 1998), and is often used as a reference point for entrepreneurial decision making (Busenitz et al., 2003). Given that these future sales aspirations are not normally distributed, we use the log of sales instead.

#### Control Variables

Demographics – such as age, education level, locus of control, risk-taking propensity, previous experience, and whether one’s parents were entrepreneurs – have been thought by other scholars to have relationships with the entrepreneurship process (Gartner, 1985). We therefore controlled for these factors in our analyses. Measures for the control variables of risk-taking propensity and innovation appear in Supplementary Appendix A, along with the coding for the other control variables.

## Results

Supplementary Appendix B contains the basic descriptive statistics and correlations for all variables.

Table 1 reports the regression results with the number of ideas as dependent variable. Recall that the number of ideas considered was not normally distributed; they are counts with large outcomes being rare. Hence, we analyzed these data using Poisson regression because such models are best used for modeling events where the outcomes are counts with non-negative integer values that count something (Neter et al., 1996). The SAS generalized linear model procedure GENMOD was used to estimate the parameters. The procedure fits models using maximum likelihood estimation. One important feature of this procedure is that the estimation allows the specification of a scale parameter to fit over-dispersion of Poisson distribution (variance greater than mean), which is a concern using Poisson regression (Long, 1997). We set the scale parameter as fixed in the model.

**TABLE 1:** Poisson regression results: Motivations and wealth aspiration on number of ideas considered.

	**Model 1 (Control)**		**Model 2 (Main effect)**		**Model 3 (Interaction)**	
**Variable**	**Coefficient**	**Std error**	**Coefficient**	**Std error**	**Coefficient**	**Std error**
Age	−0.07**	0.02	−0.06**	0.03	−0.05*	0.03
Sex	0.35**	0.08	0.25**	0.08	0.25**	0.09
Control	–0.02	0.06	0.03	0.07	0.04	0.07
Degree	0.06	0.06	0.00	0.06	–0.01	0.06
Child	0.26**	0.09	0.30**	0.09	0.30**	0.09
Employed	0.06	0.07	0.08	0.07	0.09	0.08
Busibefo	−0.25**	0.08	−0.23**	0.08	−0.21**	0.08
Parent	0.14 +	0.08	0.14 +	0.08	0.16*	0.08
Innovate	0.00	0.07	0.07	0.08	0.04	0.08
Risktake	0.14 +	0.07	0.14	0.08	0.11	0.08
Logsale5			0.05 +	0.03	0.29	0.26
Achieve			0.03	0.02	−0.70**	0.23
Independ			−0.08**	0.02	−0.35*	0.18
Willing			0.05	0.04	−0.62 +	0.36
Sale_achieve					0.06**	0.02
Sale_indep					0.03*	0.01
Sale_willing					0.05 +	0.03
Model χ^2^	385.85**		316.84**		292.70**	
-2 Log Likelihood	309.82**		340.86**		364.38**	
Δχ^2^			31.04**		23.52**	

*n* = 202. +*p* < 0.10, *p < 0.05, **p < 0.01. Constants omitted to save space.

Model 1 in Table 1 is the base model with all the control variables. The significant negative coefficients indicate that nascent entrepreneurs who search for fewer ideas tend to possess more venture experience and to be older. The significant positive coefficients indicate that entrepreneurs who search for more ideas tend to females or to be entrepreneurs with children. These findings for control variables confirm some previous findings. For example, Cooper et al. (1995) found that experienced entrepreneurs search for less information, perhaps because they can screen out losing ideas quickly (Bhide, 1994).

Model 2 in Table 1 tests hypotheses 1 through 4. Hypothesis 1 predicted that need to be independent would be positively related to the number of ideas considered. Results indicate an opposite, negative relationship: the more that these nascent entrepreneurs wanted to be independent, the less intensive they searched for an idea and the fewer ideas that they considered. Hypotheses 2 and 3 predicted that entrepreneurs with a strong need for achievement and strong willingness to become entrepreneurs would be positively related to the number of ideas that they considered. Our results do not corroborate these hypotheses.

Hypothesis 4 predicted that nascent entrepreneurs’ expectations about one’s enterprise would be positively related to the number of ideas considered, and this hypothesis receives some support (*p* < 0.10). Those who aspire to become the next millionaire or billionaire tend to consider more ideas. A billionaire-want-to-be needs to select among many ideas to find an idea so as to realize the dreams, while a person who feels comfortable with earning just enough money to support the family can more easily find an idea to realize that more constrained goal (Bhide, 1994).

**FIGURE 1:**
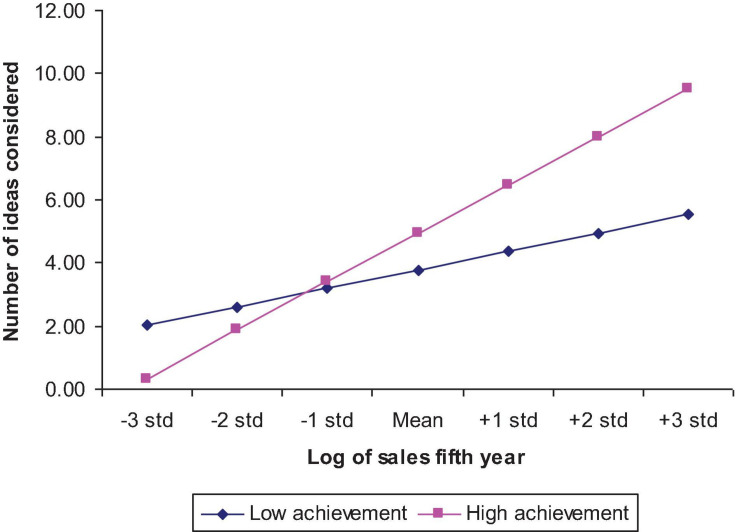
Moderating effects of need for achievement on the relationship between number of ideas considered and wealth aspiration level.

Model 3 in Table 1 tests the Hypothesis 6 prediction that the relationship between aspiration level and number of ideas considered would be moderated by entrepreneurial motivations. Empirical support for this hypothesis is evidenced by the significant interaction terms of wealth aspiration level with all three motivation variables: need to become independent, need for achievement, and willingness to become an entrepreneur. Highly motivated entrepreneurs tend to consider more ideas than less motivated ones as aspiration level increases. Figure 1 graphically depicts this relationship for the need for achievement motivation factor. The log-of-sales-in-the-fifth-year-after-initial-operation variable, representing aspiration level, is displayed in the form of standard deviations, as suggested by Florin et al. (2003). Graphs for the other motivation factors are similar.

We conducted a logistic regression analysis to test Hypotheses 5 and 7, given the dichotomous nature of the satisficing dependent variable (Gong, 2003). Model 1 is the base model with all the control variables. Regression coefficients indicate that only the control variable of business experience significantly relates to satisficing search. If a person had owned a business before, he/she will tend to select an idea more quickly and act upon it sooner than someone lacking experience. Past experience helps entrepreneurs know what to do and how to do certain things faster. For example, they know better how to get external funds and they know what products customers need and hence they tend to come out with a prototype faster (Cooper et al., 1995).

Model 2 in Table 2 tests the Hypothesis 5 prediction that aspiration level relates negatively with satisficing search. Results indicate that wealth aspiration, measured by log sales, significantly adds (*p* < 0.01) to the explained variance when compared with the control variable only model. This finding strongly supports Hypothesis 5. Entrepreneurs with high aspiration levels about their enterprises tend to narrow down their ideas slowly and they tend to delay on committing to non-retrievable investments.

**TABLE 2:** Logistic regression results of motivations and wealth aspiration on satisficing search.

	**Model 1 (Control)**		**Model 2 (Main effect)**		**Model 3 (Interaction)**	
**Variable**	**Coefficient**	**Std error**	**Coefficient**	**Std error**	**Coefficient**	**Std error**
Age	–0.01	0.09	–0.03	0.09	–0.05	0.10
Sex	–0.06	0.30	–0.10	0.32	–0.19	0.33
Control	0.23	0.23	0.43	0.27	0.44	0.28
Degree	–0.10	0.21	–0.09	0.22	–0.12	0.23
Child	0.21	0.33	0.14	0.34	0.06	0.37
Employed	0.03	0.26	0.03	0.27	0.02	0.28
Busibefo	–0.14	0.29	–0.16	0.30	–0.15	0.30
Parent	0.25	0.29	0.31	0.30	0.15	0.31
Innovate	–0.04	0.25	–0.06	0.27	–0.03	0.27
Risktake	0.30	0.24	0.33	0.25	0.35	0.26
Logsale5			−0.24*	0.12	–0.25	1.02
Achieve			0.02	0.09	0.11	0.90
Independ			–0.05	0.07	0.54	0.71
Willing			0.03	0.14	4.00*	1.52
Sale_achieve					–0.01	0.07
Sale_indep					0.04	0.05
Sale_willing					−0.31*	0.12
Wald χ^2^	7.32		13.32 +		18.81 +	
−2 Log Likelihood	278.08		263.99		250.32	
Δχ^2^			14.09*		13.67*	

*n* = 202. +*p* < 0.10, *p < 0.05, **p < 0.01. Constants omitted to save space.

Model 3 in Table 2 tests the Hypothesis 7 prediction that the negative relationship between satisficing and wealth aspiration is moderated by entrepreneurial motivation. The analysis indicates that the hypothesis received strong support overall (the change in −2 log likelihood addition is significant at *p* < 0.05). However, only the interaction involving willingness to become an entrepreneur significantly adds more explained variance to the main effect model (*p* < 0.05). Thus, Hypothesis 7 received partial support.

**FIGURE 2:**
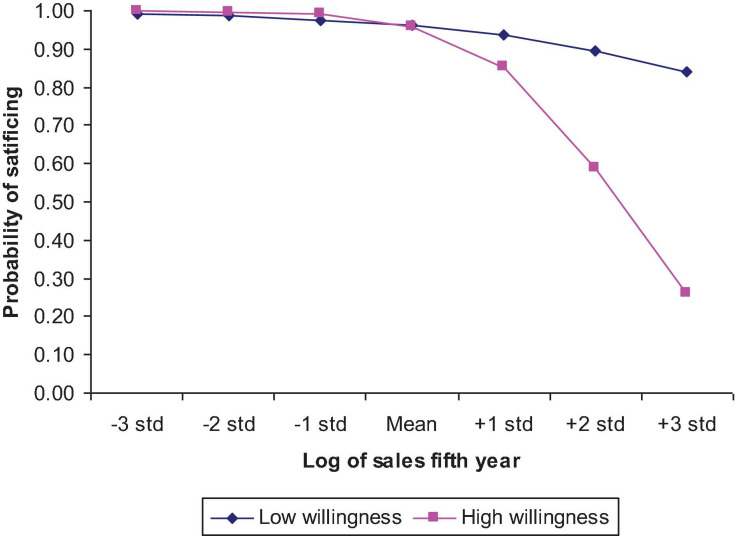
Interaction of willingness to become entrepreneurs and wealth aspiration level on probability of satisficing *. *Probability is calculated by using exp(y)/{1 + exp(y)} while *y* = constant – 0.14*logsale5 + 4.00*willingness –0.31*logsale5*achievement. We set willingness as 3.15, i.e., one STD (1.17) above the mean (1.98) and low achievement as 0.81 one STD below the mean. Constant is set to 5.

Figure 2 graphically depicts this interaction relationship. Nascent entrepreneurs who possess relatively lower willingness to become entrepreneurs tend to satisfice anyway. In such a case, their aspiration does not seem to matter much. Because their willingness is lower, they do not go out to search for opportunities, they tend to wait for the opportunities to come to them and, once an idea does come to them, they quickly take action. For highly motivated entrepreneurs, the probability of satisficing is high when their wealth aspiration is low. In such a case, their active search can yield many desirable ideas quickly and their action should be quick. But when they are highly motivated and have high aspiration levels, they will actively search for opportunities, yet it will take time to find a desirable opportunity to meet the high aspiration level. Overall, the probability of satisficing decreases when aspiration level increases. This means that highly motivated people with high aspiration are very unlikely to satisfice.

## Discussion

We examined two important aspects of nascent entrepreneurs’ searching behavior: the number of ideas considered in a 3-month period, which we labeled active search, and the degree to which they committed quickly to an idea, which we labeled satisficing search. We argued that some entrepreneurs tend to emphasize thinking, reflection, and analysis before taking action (Weick, 1983). They search for ideas in more of a planning mode (Mintzberg, 1973). These entrepreneurs are optimizers, because they use systematic searching techniques to look for the very best ideas to act upon.

Other entrepreneurs tend to take action immediately upon finding a feasible idea. This type of entrepreneur emphasizes action more than analysis in the opportunity finding process, acting on intuition, guts, and instinct. They are in the entrepreneurial mode, even when searching for ideas (Mintzberg, 1973). These entrepreneurs are satisficers, who emphasize relatively few ideas and quick action.

These two searching styles differ in their balance between the exploration of new ideas and exploitation of a chosen idea. And indeed, we found through our hypothesis testing that nascent entrepreneurs do appear to vary consistently in their balance between idea exploration and exploitation (March, 1991), based in part on their wealth aspirations and their motivation to become an entrepreneur. Consistent with our hypotheses, we found that aspiration level is positively associated with the number of ideas considered (exploration), but negatively related to satisficing (exploitation). Surprisingly, however, motivation as represented by the need for independence is negatively related to the number of ideas considered in our sample. Thus, on the one hand, high aspiration levels lead nascent entrepreneurs to search for more new idea alternatives while, on the other hand, motivation to become an entrepreneur pushes them to select an idea quickly and act upon it quickly. It may be that these potential entrepreneurs are so anxious to be independent that they act quickly, or it may be that they feel they must act immediately, before the opportunity window closes. Past research inconsistencies concerning entrepreneurial motivation (e.g., Baum et al., 2001) may be because motivation matters differently in different stages of the entrepreneurship process. These are interesting issues for future research.

We further proposed that motivation alters the relationship between aspiration level and opportunity search behavior. Our findings indicate that the need to be independent, the need for achievement, and willingness to become an entrepreneur all affect the relationship between wealth aspiration level and the number of ideas considered. When motivations are high, entrepreneurs consider more and more ideas as aspiration increases. When motivations are low, entrepreneurs consider fewer ideas regardless of aspiration levels. Thus, aspirations and motivation work jointly in influencing opportunity search.

The relationship between aspiration level and satisficing was also moderated by motivation in our sample, although only by the motive focusing on their willingness to become an entrepreneur. For highly willing potential entrepreneurs, low aspiration means that a satisfactory idea can be found easily and hence action can be taken quickly, while high aspiration mandates more wide-ranging search and therefore less satisficing. For less willing potential entrepreneurs, because they do not actively search for ideas they likely encounter only a few ideas, and these ideas are very likely to come to them as a surprise (Shane, 2000). Hence, no matter what their level of wealth aspiration, they would likely settle on the first feasible idea.

**FIGURE 3:**
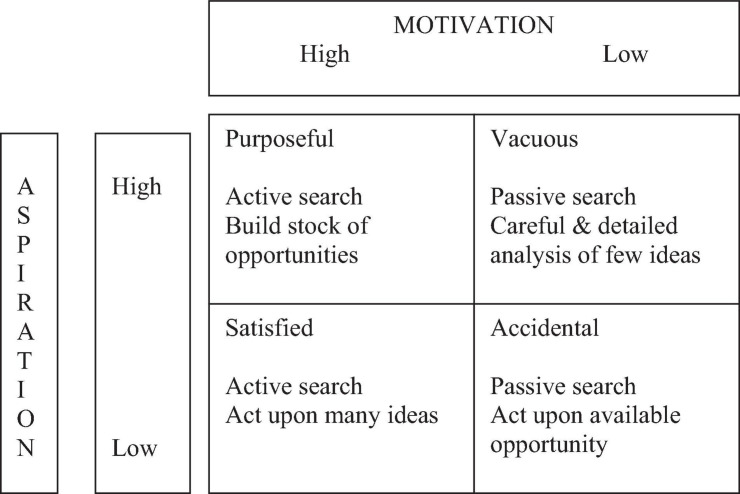
Aspiration and motivation with entrepreneurial search.

Our research makes a few theoretical and practical contributions. First., we answer recent calls to study how motivations affect opportunity search behavior (Yitshaki and Kropp, 2018; Murnieks et al., 2020). We show that studying motivation is promising provided that right motivations should be used and that interaction effect of motivations and other factors should be investigated. Second, our article helps reconcile the debate on if entrepreneurs find opportunities passively or systematically (Tang et al., 2012). Specifically, we found that aspirations may push entrepreneurs to search for opportunities actively while need for independence makes opposing impact. Third, based on our research, we propose a typology of entrepreneurial opportunity search behavior in Figure 3 that exhibits four types of nascent entrepreneurs based on the two dimensions: accidental, satisfied, vacuous, and purposeful. Government agencies who wish to encourage more entrepreneurship may use the typology to sponsor the right type of nascent entrepreneurs, purposeful entrepreneurs. They are the type that are motivated and are ready to create substantial opportunities.

Our study has several limitations. First, everyone in our sample of nascent entrepreneurs resides in the Midwest. Future research in other geographies could confirm the generalizability of our results. Second, there is some potential for common method variance due to the use of single respondents (Podsakoff et al., 2003). Our use of objective, self-report count data (i.e., the number of ideas considered) for the dependent variable, combined with the complexity of our interaction hypotheses, should combine to mitigate this concern. Social desirability bias is also a concern. Respondents could possibly exaggerate the number of ideas they considered. Our results, however, are similar to those of Ucbasaran and Westhead (2002); the mean number of ideas reported by our nascent entrepreneurs reported is 3.08, while their habitual entrepreneurs reported 2.95. This reduces the likelihood that social desirability is affecting our results. Finally, we measured aspiration level using predicted fifth year sales, which may be an indication of confidence. However, other researchers have employed similar approach to measure aspiration. For example, Puente et al. (2017) used the forecasted number of employees in the fifth year as aspiration. We suggest future research include confidence as a control variable.

Overall, our research takes a step toward a better understanding of prospective entrepreneurs’ opportunity search behaviors – an area central to entrepreneurship. Our analysis shows that nascent entrepreneurs do differ from each other in the search process, judged by the number of ideas that they consider and the quickness with which they commit resources to an idea. Moreover, we found that wealth aspirations and motivation to become an entrepreneur jointly influence the opportunity finding process. Thus, these relationships are more complex than previously thought, and much remains to be learned. We hope that our research spurs other scholars to join in this vital area of study.

## Data Availability Statement

The datasets presented in this article are not readily available because this is part of a larger dataset and it can’t be shared wholly. Requests to access the datasets should be directed to SW, sibinwu@hotmail.com.

## Ethics Statement

The studies involving human participants were reviewed and approved by the University of Wisconsin-Milwaukee. The patients/participants provided their written informed consent to participate in this study.

## Author Contributions

RZ did the work on the data collection and data analysis. SW contributed to the other work and also made sure that the manuscript reads well. Both authors contributed to the article and approved the submitted version.

## Conflict of Interest

The authors declare that the research was conducted in the absence of any commercial or financial relationships that could be construed as a potential conflict of interest.
