# Supplementary material for: Opportunity Finding by Nascent Entrepreneurs: Accidental or Purposeful?
Source: Front Psychol. 2021 Jan 28;11:592994. doi: 10.3389/fpsyg.2020.592994 (PMC7875887; doi:10.3389/fpsyg.2020.592994)
Supplement: Supplementary file 1 [file Data_Sheet_1.DOC]

**Appendix A**

Variables and their Descriptions

| Variable | Description | Values |
| --- | --- | --- |
| Ideas | # of Ideas considered in three months | From 1 to 40 |
| Satisfice | Ideas<=2, select one & three activities | 1=satisfice, 0=non-satisfice |
| Willing | Sum of 4 items from Mitchell (2000) | From 0 to 4 =strong desire |
| Independ | Sum of 4 items from Robichaud (2002) | From 4 to 20 |
| Achieve | Sum of 3 items from Robichaud (2002) | From 3 to 15 |
| Logsale5 | Log value of expected sale in fifth years | Continuous |
| Age | Six categories | 6 =the oldest group |
| Sex | Female and male | Male=1 |
| Control | My future is within my control | From 1 to 5=strongly agree |
| Degree | 4 categories | 1=no college 4=doctoral |
| Child | Have children or not | 1=have children |
| Employed | Employed or not | 1=employed |
| Busibefo | Created own business before | 1=yes |
| Parent | Parent(s) own business | 1=yes |
| Innovate | I like to innovate | From 1 to 5=strongly agree |
| Risktake | I am willing to take risk | From 1 to 5=strongly agree |

**Appendix B**

**Simple Statistics and Correlations***

|  | **Variable** | 1 | 2 | 3 | 4 | 5 | 6 | 7 | 8 | 9 | 10 | 11 | 12 | 13 | 14 | 15 | 16 |
| --- | --- | --- | --- | --- | --- | --- | --- | --- | --- | --- | --- | --- | --- | --- | --- | --- | --- |
| 1 | Ideas | 1.00 |  |  |  |  |  |  |  |  |  |  |  |  |  |  |  |
| 2 | Satisfice | -0.31 | 1.00 |  |  |  |  |  |  |  |  |  |  |  |  |  |  |
| 3 | Willing | 0.10 | 0.08 | 1.00 |  |  |  |  |  |  |  |  |  |  |  |  |  |
| 4 | Independ | -0.11 | 0.06 | -0.03 | 1.00 |  |  |  |  |  |  |  |  |  |  |  |  |
| 5 | Achieve | -0.02 | 0.04 | -0.07 | 0.63 | 1.00 |  |  |  |  |  |  |  |  |  |  |  |
| 6 | logsale5 | 0.14 | -0.13 | 0.06 | -0.01 | 0.06 | 1.00 |  |  |  |  |  |  |  |  |  |  |
| 7 | Age | -0.11 | 0.00 | -0.09 | -0.02 | -0.00 | -0.13 | 1.00 |  |  |  |  |  |  |  |  |  |
| 8 | Sex | 0.17 | -0.04 | 0.10 | -0.18 | -0.16 | 0.04 | -0.04 | 1.00 |  |  |  |  |  |  |  |  |
| 9 | Control | -0.02 | 0.09 | 0.09 | 0.48 | 0.45 | 0.04 | 0.02 | -0.12 | 1.00 |  |  |  |  |  |  |  |
| 10 | Degree | 0.07 | -0.03 | 0.02 | -0.10 | -0.04 | 0.07 | 0.14 | 0.01 | 0.03 | 1.00 |  |  |  |  |  |  |
| 11 | Child | 0.10 | 0.00 | 0.03 | -0.00 | -0.03 | 0.06 | -0.24 | 0.05 | -0.03 | 0.27 | 1.00 |  |  |  |  |  |
| 12 | Employed | 0.02 | -0.05 | -0.12 | 0.06 | -0.01 | 0.01 | 0.03 | 0.03 | -0.01 | 0.04 | 0.06 | 1.00 |  |  |  |  |
| 13 | Busibefo | -0.09 | -0.03 | -0.12 | -0.05 | -0.07 | 0.05 | -0.16 | 0.03 | 0.05 | 0.01 | 0.17 | 0.25 | 1.00 |  |  |  |
| 14 | Parent | 0.04 | -0.02 | -0.04 | -0.01 | 0.01 | 0.01 | 0.04 | 0.11 | 0.02 | 0.06 | 0.02 | 0.15 | 0.24 | 1.00 |  |  |
| 15 | Innovate | 0.02 | 0.06 | 0.03 | 0.47 | 0.46 | -0.04 | 0.09 | -0.14 | 0.42 | 0.13 | 0.02 | 0.17 | -0.13 | -0.05 | 1.00 |  |
| 16 | Risktake | 0.08 | 0.10 | 0.12 | 0.46 | 0.38 | 0.06 | -0.08 | -0.04 | 0.46 | -0.06 | -0.04 | 0.16 | -0.14 | -0.09 | 0.60 | 1.00 |
|  |  |  |  |  |  |  |  |  |  |  |  |  |  |  |  |  |  |
|  | Mean | 3.08 | 0.51 | 1.98 | 16.97 | 13.32 | 12.93 | 3.79 | 1.37 | 4.61 | 1.70 | 1.37 | 1.37 | 1.74 | 1.77 | 4.30 | 4.15 |
|  | Std | 3.58 | 0.50 | 1.17 | 2.97 | 2.24 | 1.31 | 1.74 | 0.48 | 0.78 | 0.74 | 0.48 | 0.59 | 0.56 | 0.50 | 0.81 | 0.89 |

* Absolute values between 0.111 and 0.141 are significant at *p*<.10, between 0.141 and 0.171 at p<0.05, and greater than 0.171 at p<0.01. n = 230
